# Histologic and Metabolic Derangement in High-Fat, High-Fructose, and Combination Diet Animal Models

**DOI:** 10.1155/2015/306326

**Published:** 2015-05-18

**Authors:** Jai Sun Lee, Dae Won Jun, Eun Kyung Kim, Hye Joon Jeon, Ho Hyun Nam, Waqar Khalid Saeed

**Affiliations:** ^1^Department Translational Medicine, Graduate School of Biomedical Science and Engineering, Hanyang University, Seoul, Republic of Korea; ^2^Department of Internal Medicine, School of Medicine, Hanyang University, Seoul, Republic of Korea; ^3^Department of Pathology, College of Medicine, Eulji University, Seoul, Republic of Korea

## Abstract

*Background*. We used high-fat (HF), high-fructose (HFr), and combination diets to create a dietary animal model of nonalcoholic fatty liver disease (NAFLD). Comparison of both clinical phenotypes has not been well defined. The purpose of this study was to compare histologic and metabolic characteristics between diets in an animal model of NAFLD. *Methods*. NAFLD was induced in rats by feeding them HF, HFr, and combination (HF + HFr) diets for 20 weeks. The degree of intrahepatic fat accumulation, inflammation, and oxidative stress was evaluated. Metabolic derangements were assessed by the oral glucose tolerance test and the intrahepatic insulin signal pathway. *Results*. Body weight gain and intrahepatic fat accumulation were more prominent in the HF feeding group than in the HFr group. The expressions of NOX-4 and TLR-4 were higher in the HF and HFr combination groups than in the HF-only group. Other intrahepatic inflammatory markers, MCP-1, TNF-*α*, and endoplasmic reticulum stress markers, were the highest in the HF + HFr combination group. Although intrahepatic fat deposition was less prominent in the HFr diet model, intrahepatic inflammation was noted. *Conclusions*. Intrahepatic inflammation and metabolic derangements were more prominent in the HF and HFr combination model than in the HF monodiet model.

## 1. Introduction

Nonalcoholic fatty liver disease (NAFLD) is the most common form of chronic liver disease in western countries. The underlying mechanisms leading to NAFLD progression and serious consequences such as cirrhosis and hepatocellular carcinoma are still largely unknown [1]. There are two forms of fatty liver disease, simple steatosis and steatohepatitis. In some cases, steatohepatitis, not simple steatosis, leads to cirrhosis, hepatocellular carcinoma, and end-stage liver disease [2].

A number of genetic and dietary NAFLD models are used to understand NAFLD pathophysiology; however, in practice, only a few models successfully achieve inflammatory steatohepatitis and fibrosis [1]. One of the most challenging aspects of NAFLD studies is the lack of a suitable animal model for steatohepatitis and NASH-associated fibrosis [1]. An ideal NASH animal model should closely resemble the features of human NASH and include features such as obesity, insulin resistance, and histological changes including liver inflammation and fibrosis. However, an ideal NASH model that closely mimics both human clinical and histological features has not been established. For instance, although the methionine- and choline-deficient- (MCD-) induced malnutrition model closely resembles the histological features of human steatohepatitis, the animals rarely develop insulin resistance or obesity [3]. The pathophysiological mechanism is also quite different from that in actual clinical settings. Animal models with ad libitum feeding of 40–60% high-fat (HF) diets successfully develop obesity, intrahepatic fat deposition, insulin resistance, and metabolic parameters resembling human NASH [4].

Recent epidemiological studies have highlighted a strong correlation between intrahepatic fat accumulation and saturated fats, trans-fatty acids, carbohydrates, and simple sugars. However, the true association with specific nutrients still remains unclear [5]. Both human and animal studies have established the impacts of fructose consumption on body weight, lipid profiles, and glucose metabolism. It is well documented that fructose-enriched diets also contribute to an increased risk of developing obesity, metabolic syndrome, and diabetes mellitus [6, 7]. However, until now, only a few studies have compared the histological and metabolic alterations induce by HF, HFr, and HF + HFr diets in order to examine the different NAFLD and/or NASH in animal models [8]. Therefore, we evaluated and compared the differences in the induction of histological and metabolic features induced by HF, HFr, or HF plus HFr combination diets to characterize the resulting NAFLD and NASH rat models.

## 2. Materials and Methods

### 2.1. Experimental Design

Forty-eight male Sprague-Dawley rats (Orient Bio, Seoul, Korea) (200–250 g) were randomly allocated to four groups as follows: control (*n* = 12) fed on normal chow, high-fat diet (*n* = 12) fed on a high-fat diet (60% total calories), high-fructose (*n* = 12) (30% fructose in drinking water), and high-fat + high-fructose combination (*n* = 12) (60% high fat + 20% fructose). Fructose (20–30%) was only supplied in drinking water. Animals were maintained in a temperature-controlled room (22°C) on a 12:12 h light-dark cycle. During the 20-week period, changes in body weight were recorded weekly and at the end of the experiment. The study was designed to execute three separate sets of experiments each at end of 12, 16, and 20 weeks. At 12, 16, and 20 weeks, 4 rats from each group were sacrificed by thoracotomy involving the diaphragm. Blood was withdrawn by cardiac puncture, and liver tissues were extracted for evaluation. All procedures were approved by the Hanyang Institutional Animal Care and Use Committee (HY-IACUC-11-067).

### 2.2. Oral Glucose Tolerance Test and Blood Chemistry

Oral glucose tolerance testing (OGTT) was performed at the end of weeks 12, 16, and 20 before animal sacrifice. Briefly, 2 g glucose/kg body weight was provided orally to the rats. After 0, 30, 60, and 120 minutes blood glucose was measured from tail vein blood using an Accu-Chek glucose meter (Roche, Montreal, QC, Canada). Upon animal sacrifice, the serum aspartate aminotransferase (AST) and alanine aminotransferase (ALT) levels were measured using a biochemical analytical system (Hitachi-747; Hitachi, Tokyo, Japan). Interleukin-6 (IL-6) was measured by ELISA (R&D Systems, Minneapolis, MN, USA).

### 2.3. Liver Histology and Immunohistochemistry

Paraffin-embedded liver sections were fixed in 10% formaldehyde and stained with haematoxylin and eosin. Four-micrometer-thick sections were cut from the paraffin block and coated on the glass slide. Immunohistochemistry for expression of NOX-4 (Abcam, Cambridge, UK) and F4/80 (Abcam, Cambridge, UK) was performed. A single pathologist blinded to treatment status evaluated the histological characteristics and calculated overall steatohepatitis scores. Histologic evaluation was performed using the NAFLD Activity Score (NAS) system [9].

### 2.4. RNA Isolation and Quantitative RT-PCR

RNA was isolated from the liver tissue using the RNeasy kit (Qiagen, Dusseldorf, Germany) according to the manufacturer's instructions. Isolated RNA samples were converted to cDNA using reverse transcriptase (SuperScript III; Invitrogen) and oligo (dT) primers. All PCR reactions were performed on the LightCycler 480 system (Roche Diagnostics, Mannheim, Germany) using LightCycler480 SYBRGreen I Mastermix (Roche Diagnostics) in standard 10 *μ*L reaction volumes as follows: 4 *μ*L (100 ng) cDNA, 0.5 *μ*L of 10 pM sense primer, 0.5 *μ*L of 10 pM antisense primer, and 5 *μ*L LightCycler 480 SYBR Green I Master mix (Roche Diagnostics). To guarantee the reliability of the obtained results, all samples were processed in triplicate, and each assay was performed using a negative control. The values thus obtained were normalized versus the control and were expressed as fold changes. PCR primer sets used were as follows: TNF-a forward, 5′-TGAGATTCGTGCACAAGAGG-3′; reverse, 5′-GTCATGGCTTTGGATGTCCT-3′; MCP-1 forward, 5′-ATGCAGTTAATGCCCCACTC-3′; reverse, 5′-TTCCTTATTGGGGTCAGCAC-3′; CHOP forward, 5′-GCAGCTGAGTCTCTGCCTTT-3′; reverse, 5′-CTGCTCCTTCTCCTTCATGCTTCCCCGTTCT-3′; ATF6 forward, 5′-CCCACCAAAGGTCAGACTGT-3′; reverse, 5′-CTTGGGACTTTGAGCCTCTG-3′; IRS-1 forward, 5′-ACACAGCTGCACAGACCAAC-3′; reverse, 5′-CCCAACTCAACTCCACCACT-3′; IRS-2 forward, 5′-CATCGATGGCCTTCTCTCTC-3′; reverse, 5′-CCATGAGACTTAGCCGCTTC-3′; GAPDH forward, 5′-TGCCACTCAGAAGACTGTGG-3′; reverse, 5′-TTCAGCTCTGGGATGACCTT-3′. All experiments were performed in triplicate.

### 2.5. Western Blot Analysis

PBS washed liver samples were solubilized in radioimmunoprecipitation assay buffer containing protease inhibitors (Pierce, Rockford, IL). The lysates were centrifuged (15,000 ×g for 10 min at 4°C), and supernatants were boiled with sodium dodecyl sulfate buffer (0.5 M *β*-mercaptoethanol). The boiled lysates were subjected to 10% SDS-PAGE, and signals were visualized using enhanced chemiluminescence western blot analysis detection reagent (Amersham Biosciences, Piscataway, NJ). The bands on immunoblots were analyzed using an image reader LAS-3000 (version 2.1; Fujifilm, Tokyo). The primary antibodies, CHOP and ATF-6 polyclonal antibodies (Santa Cruz Biotechnology, Inc., Santa Cruz, CA, USA), were diluted 1 : 200 in TBS-T/5% nonfat dry milk, and the secondary antibody was horseradish peroxidase- (HRP-) conjugated goat anti-mouse IgG antibody diluted 1 : 2000 (Santa Cruz Biotechnology, Inc.) in TBS-T/5% powdered nonfat dry milk. This experiment was repeated in three samples.

### 2.6. Data Analysis

All quantitative data were expressed as the group means and standard deviations. Statistical analysis was performed with the one-way ANOVA test using SPSS for Windows version 18.0 (SPSS Inc., Chicago, IL, USA). *p* values less than <0.05 were considered significant (see Table 1).

## 3. Results

### 3.1. Baseline Characteristics and Biochemical Analysis

**Figure 1:**
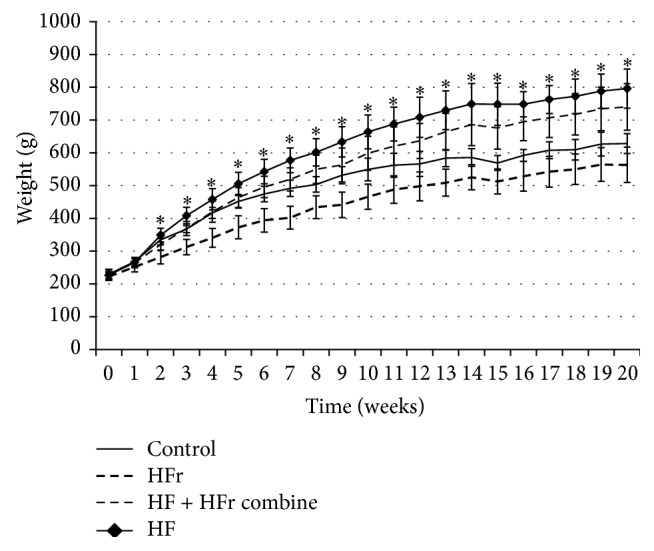
Changes in body of rats fed control, high-fat, high-fructose, and combination diet groups for the indicated weeks ^∗^ *p* < 0.05 by ANOVA.

At baseline, the body weights of the rats were the same. Compared to the control at 12, 16, and 20 weeks, the body weight of rats in the HF diet (796 ± 69.74 g) and HF + HFr diet (740.2 ± 65.43 g) groups increased significantly (*p* = 0.01 and *p* = 0.03, resp.), while body weight of rats in the HFr group decreased; this decrease was not significant (*p* = 0.122) (Figure 1). Among all of the groups, differences in serum aspartate aminotransferase (AST), alanine aminotransferase (ALT), total bilirubin, fasting insulin, and IL-6 levels were found; however, none of these differences were significant (data not shown).

### 3.2. Histological Findings

**Figure 2:**
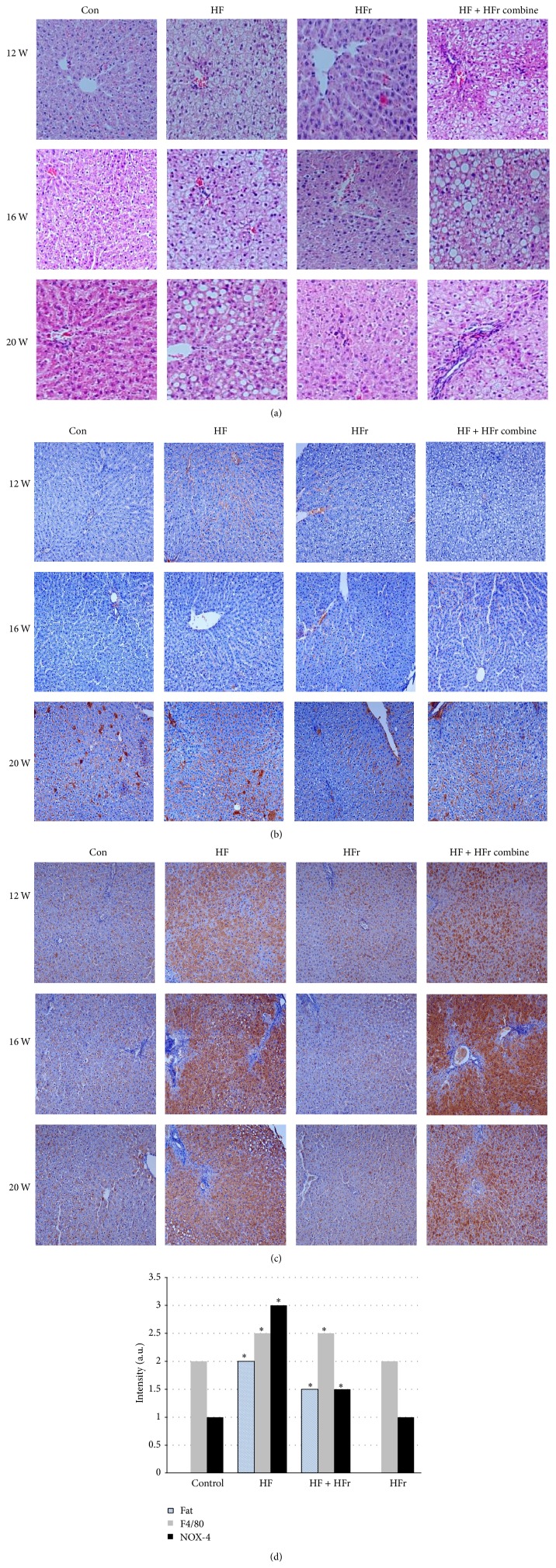
Nonalcoholic fatty liver disease caused by high-fat and high-fructose diet in rats. Histological examination of liver sections by hematoxylin and eosin staining (a) and immunohistochemistry F4/80 (b), NOX-4 (c), and TLR-4 (d).

H&E staining demonstrated a progressive development of steatosis in both HF and HF + HFr groups (Figure 2). Moreover, compared to the control, the HF group showed a significant increase in intrahepatic fat deposition, while the HFr group showed only minimal fat deposition (Figure 2(a)). F4/80 and Nox-4 immunohistochemistry expressions were measured to evaluate intrahepatic inflammation. Tissues from the HF and HF + HFr groups stained more strongly with F4/80 than those from the HFr group, especially at 20 weeks (Figure 2(b)). Nox-4 was also significantly increased in the HF and HF + HFr groups compared to the control group (*p* = 0.05) (Figure 2(c)).

### 3.3. Metabolic Derangement and Glucose Tolerance Test

**Figure 3:**
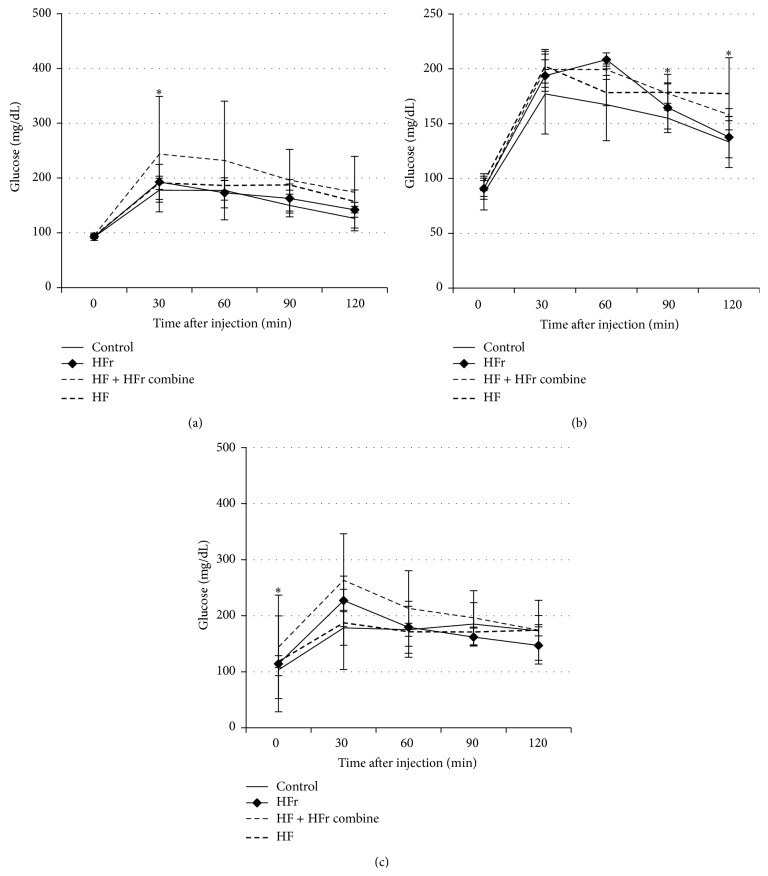
Effect of control, high-fat, high-fructose, and combination diet groups on insulin sensitivity. Blood glucose levels during the oral glucose tolerance test: 12 weeks (a); 16 weeks; (b) 20 weeks (c). ^∗^ *p* < 0.05 by ANOVA.

An oral glucose tolerance test (OGTT) was conducted at 12, 16, and 20 weeks (Figure 3). Only rats in the HF + HFr group showed an area under the curve that was higher than the control group at 12 weeks and 20 weeks. Receiver operating characteristic values of the fructose group were significantly higher than the other groups (*p* = 0.041). Compared to the control at 12, 16, and 20 weeks, the serum glucose levels increased significantly (*p* = 0.05) in rats fed the HF + HFr diet (Figure 3).

### 3.4. Intrahepatic Inflammation and Endoplasmic Reticulum Stress

**Figure 4:**
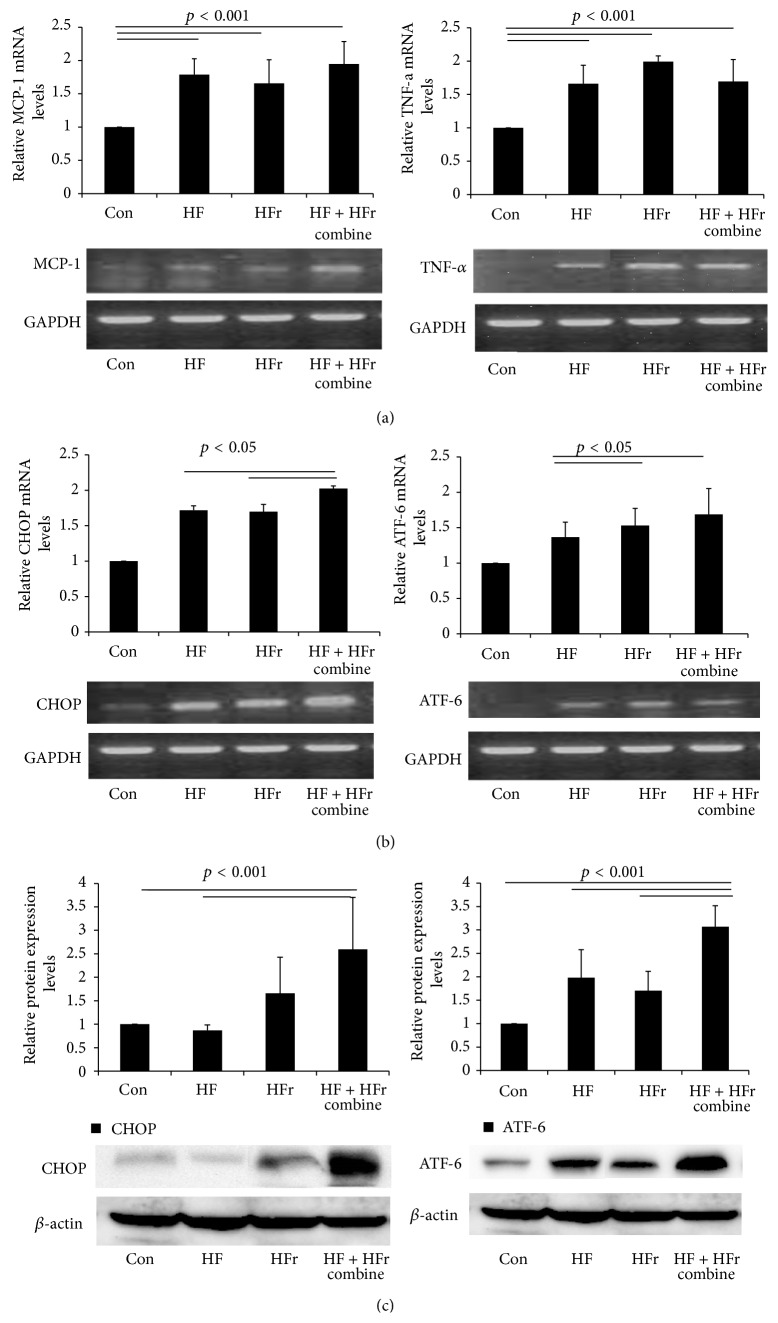
Changes in hepatic expression of genes involved in endoplasmic reticulum stress, inflammation, and insulin resistance. Gene expression of inflammation markers in the liver from each group was investigated by RT-PCR analysis (a). Detection of endoplasmic reticulum stress markers in the liver from each group using RT-PCR (b). Detection of CHOP and ATF-6 in the liver from each group using western blotting (c). *N* = 3  ^∗^ *p* < 0.05 by ANOVA.

To evaluate the proinflammatory status of each study group, we evaluated the mRNA expression of tumor necrosis factor-*α* (TNF-*α*) and monocyte chemoattractant protein-1 (MCP-1) using RT-PCR (Figure 4(a)). Compared to the control, the TNF-*α* and MCP-1 expression levels from the HF, HFr, and HF + HFr groups increased significantly; however, there was no significant difference among the treatment groups themselves. The C/EBP homologous protein (CHOP) and activating transcription factor 6 (ATF6) expression levels were evaluated to assess endoplasmic stress. CHOP and ATF6 expressions increased in all treatment groups compared to the control (Figure 4(b)). CHOP and ATF6 mRNA expressions were higher in the HF + HFr group than in the HF alone group. The protein expression of the ER stress marker showed similar results (Figure 4(c)).

## 4. Discussion

In our modern culture, corn syrup has emerged as a risk factor for metabolic derangement [10]. Fructose, the major ingredient of corn syrup, is known to have a strong correlation with obesity as well as brain cognitive function. Fructose promotes not only hepatic steatosis and intestinal bacterial overgrowth but also hepatic inflammation through augmented gut endotoxin-mediated Kupffer cells activation [11].

Our data showed that endoplasmic reticular stress and metabolic derangement were augmented in rats fed a combination high-fructose and HF diets. In previous studies, most HF diets contained 45–60% fat, and rodents fed these HF diets had up to a 15% increase in body weight due to the gradual accumulation in body fat. Interestingly, mixing 30% HFr with drinking water did not significantly increase total body weight compared to the normal chow diet group. However, HFr feeding significantly augmented intrahepatic inflammation when cotreated with the HF diet. Hepatic expressions of F4/80 and NOX4 were more prominent in the HF + HFr combination diet group than in the HFr and HF alone groups, suggesting that fructose augments hepatocellular damage. Notably, feeding with fructose alone did not cause the rats to fully express the phenotype of human NASH or fibrosis. These results are similar to human data. In a human study, fructose consumption was significantly higher in NASH patients than in simple steatosis patients independent of body weight, age, and sex [12]. Moreover, daily fructose consumption is associated with increased hepatic fibrosis [13, 14]. There are some possible mechanisms by which fructose can act as a facilitator of inflammation and fibrosis. Fructose increases the influx of lipopolysaccharides from the gut by changing small intestine bacterial overgrowth and intestinal permeability [15]. This increase in lipopolysaccharides induces intrahepatic innate immunity and ROS production [16, 17]. Additionally, the HFr diet not only increases insulin resistance but also promotes mild-to-severe dyslipidemia [18]. One report insisted that fructose can induce diabetes by stressing pancreatic islet cells and depleting intracellular adenosine triphosphate [19]. In a human epidemiologic study, the amount of fructose consumption showed a strong positive correlation with the development of insulin resistance and obesity [20]. The HFr diet is a well-recognized model for metabolic syndrome. However, only a few studies have compared HF- and HFr-induced intrahepatic inflammation and oxidative stress. Kawasaki et al. compared the histological features of HF- and HFr-induced animal NAFLD models [8]. They reported that HFr diet significantly increased macrovesicular steatosis and hepatic triglycerides contents [8]; however, the extent of hepatic inflammation was not compared. Currently, there is no direct comparing data regarding the differences in diet-induced NAFLD models.

It is still not clear whether fructose alone can alter metabolic parameters. In our study, we did not find any differences in glucose intolerance between the HF and HFr groups, but glucose intolerance was significantly increased in the HF + HFr combination group. These findings are consistent with Tetri et al. [21] who determined that fructose along with the percentage of trans-fats or high fats not only promotes glucose and insulin insensitivity but also enhances fatty liver progression. Our study showed that consuming a conventional HF diet for 20 weeks successfully induced relevant simple steatosis in rats. HFr feeding supplied in drinking water successfully increased ER stress and intrahepatic inflammatory signals; however, the gains in body weight and intrahepatic fat deposition in these animals were not significant. de Moura et al. pointed towards the limitation of fructose supplied in drinking water [22]; they suggested that the HFr diet effectively induces higher insulin resistance and weight gain compared to fructose supplied in drinking water [22]. We found that HFr supplied in drinking water with a HF diet successfully induced both intrahepatic fat deposition and inflammation. In conclusion, the HF + HFr combination diet successfully augments intrahepatic inflammation and metabolic derangement compared to HF or HFr diet alone.

## Figures and Tables

**Table 1:** Biochemical parameters according to nonalcoholic fatty liver disease model.

Parameter	Control	HF	HF + HFr combination	HFr	*p* ^∗^
AST (U/L)	143 ± 25.5	336 ± 108.3^∗^	230.3 ± 78^∗^	240 ± 83.9	0.035
ALT (U/L)	44 ± 4.6	60.7 ± 22.4^∗^	48.9 ± 12.9^∗^	40.7 ± 8.7	0.049
T.bili (mg/dL)	0.02 ± 0.01	0.06 ± 0.03	0.03 ± 0.01	0.04 ± 0.02	0.121
IL-6 (pg/mL)	13.1 ± 6.1	10.3 ± 5	9.3 ± 7.9	11.3 ± 7.5	0.127
Insulin (pmol/L)	1,181 ± 1020.4	2,092.3 ± 1113.4	1,518.5 ± 1217.5	1,227.8 ± 364.2	0.513

AST: aspartate transaminase; ALT: alanine transaminase; T.bili: total bilirubin; IL-6: interleukin-6. ^∗^ *p* < 0.05 by Kruskal-Wallis test.
